# Paving the Way for Synthetic Intrinsically Disordered Polymers for Soft Robotics

**DOI:** 10.3390/polym15030763

**Published:** 2023-02-02

**Authors:** Orkid Coskuner-Weber, Elif Yuce-Erarslan, Vladimir N. Uversky

**Affiliations:** 1 Molecular Biotechnology, Turkish-German University, Sahinkaya Caddesi, No. 106, Beykoz, Istanbul 34820, Turkey; 2 Chemical Engineering, Istanbul University-Cerrahpasa, Avcılar, Istanbul 34320, Turkey; 3 Department of Molecular Medicine, Morsani College of Medicine, University of South Florida, Tampa, FL 33612, USA

**Keywords:** synthetics intrinsically disordered polymers (sIDPs), soft robotics, design, synthesis, characterization

## Abstract

Nature is full of examples of processes that, through evolution, have been perfected over the ages to effectively use matter and sustain life. Here, we present our strategies for designing intrinsically disordered smart polymers for soft robotics applications that are bio-inspired by intrinsically disordered proteins. Bio-inspired intrinsically disordered smart and soft polymers designed using our deep understanding of intrinsically disordered proteins have the potential to open new avenues in soft robotics. Together with other desirable traits, such as robustness, dynamic self-organization, and self-healing abilities, these systems possess ideal characteristics that human-made formations strive for but often fail to achieve. Our main aim is to develop materials for soft robotics applications bio-inspired by intrinsically disordered proteins to address what we see as the largest current barriers in the practical deployment of future soft robotics in various areas, including defense. Much of the current literature has focused on the de novo synthesis of tailor-made polymers to perform specific functions. With bio-inspired polymers, the complexity of protein folding mechanisms has limited the ability of researchers to reliably engineer specific structures. Unlike existing studies, our work is focused on utilizing the high flexibility of intrinsically disordered proteins and their self-organization characteristics using synthetic quasi-foldamers.

## 1. Introduction

Body parts, and/or in some cases, the entire robot, consist of a continuously deformable structure in soft robots, which is usually made from elastomeric polymers including polyurethane, and which we use differently [1]. A large number of degrees are associated with soft body parts, leading to a large-scale deformation mode [2]. In fact, flexible devices are actuated through variable tendons which can be integrated through tension cables, shape memory polymers, or shape memory alloy cables, or they are pneumatically driven by placing their internal fluidic channels and chambers under vacuum or under pressure [3]. Soft robots are suitable for applications in dynamic and uncertain and task environments and for safe human–robot interactions [4]. Additionally, due to their high degree of compliance, soft actuators adapt their shape when in contact with an object [5].

Despite the many advantages of soft materials, their usage in robotics presents a challenge in terms of damage resistance [6]. Because of the flexibility of currently available synthetic materials, soft robots are prone to damaging modes, meaning that their parts are susceptible to tears, cuts, and perforations caused by sharp objects present in the environment in which the robots operate, which in turn limits their lifespan [7]. When actuating soft robotics using tendon mechanisms, the hard tendons, which are in many cases polyamine or Kevlar wires, can cut through the soft parts [8]. This tendon cut can occur gradually due to abrasive damage resulting from repetitive friction, or upon overloading the actuators [9]. A large part of the soft robot’s body undergoes cyclic deformation upon operation, and the parts are susceptible to fatigue; micro damages due to cyclic loading may propagate into macroscopic damages and failure of parts/components [9].

Furthermore, when overloading a soft robotic component, the induced stress may exceed the mechanical strength of the polymer, which in turn can lead to rupture of the robot’s body [9]. Many soft robots are produced through molding or casting techniques. [8]. During a molding or casting step, the prepolymer crosslinks and solidifies in a mold [10]. Parts made in different molding steps are joined by an adhesive or some uncured prepolymer [11]. This multistep manufacturing technique introduces weak material interfaces that rely on secondary interactions rather than covalent bonds [12]. Upon extensive loading or multiple actuation cycles, these weak interfaces fail and interfacial debonding occurs [12]. This process is known as delamination, which leads to leaks and reduced performance [12]. Usage of organic materials leads to soft robots suffering from photodegradation due to UV radiation [12].

In biological systems, forms and functions are intertwined: properly designed structural mimics of a molecule will often be able to perform similar function(s) [13]. While protein engineers are making strides in designing de novo tailor-made proteins to perform specific functions, the complexity of protein folding mechanisms has limited the ability to reliably engineer a specific tertiary structure, even for an average-size protein [14]. As an alternative approach, in circumventing the “protein folding problem”, there is growing interest in recapitulating protein function in synthetic scaffolds or “foldamers” [15]. A simple foldamer-based biomimetic system, in which the parameters can be precisely and independently controlled, is well-suited for interrogating structure–activity relationships [16].

Many types of foldamer-based scaffolds have been found to mimic α-helical, β-sheet, or β-turn-like secondary structure elements. Examples of well-characterized foldamer scaffolds include α/β peptides, β-peptides, γ-peptides, N-substituted glycines (peptoids), phenylene ethynylenes, and urea derivatives [17]. Recently, a helix-coil foldamer was reported [18]. All these classes of non-natural oligomers have been shown to exhibit kinetic and thermodynamic stability and can mimic peptide functions [16]. While notable progress has been made in mimicking simple peptide/protein secondary structures, the prospect of creating foldamer-based supramolecular assemblies that adopt discrete tertiary and quaternary structures opens the possibility of regulating far more complex protein-like functions [19].

From a biological point of view, intrinsically disordered proteins (IDPs) are proteins that lack fixed or ordered three-dimensional structures, especially in the absence of interaction partners such as RNA, DNA, other proteins, or small molecules [20]. IDPs can be fully unstructured or partially structured and include random coil, molten globule-like conformers, or flexible linkers in large multidomain proteins [21]. IDPs have been identified to participate in weak multivalent interactions that are highly cooperative and dynamic, which make them crucial in signaling [22]. Some IDPs can adopt at least a partially ordered structure after binding to partners [23]. All in all, IDPs differ from structured proteins in many ways and tend to have distinctive functions, structures, sequences, interactions, evolution, and regulation [24].

In the 2010s, it became clear that IDPs are common among disease-related proteins [25,26]. We have been investigating the structures and the roles of amino acid residues on the structural properties and conformational dynamics of IDPs for more than 10 years [25,27,28,29]. Here, we propose to mimic IDPs, which usually gain function based on the ligand/partner binding and can have multifunctional properties based on ligand binding coordination chemistry variations [30,30,31,32]. They can also self-assemble, which makes them attractive targets for self-healing [33,34]. The most common approach in self-healing polymers is to use the dynamic covalent bonds as crosslinks for constructing polymer networks [9]. Networks with flexible chain segments and relatively low crosslink density possess elastomeric behavior that may be suitable for soft robotics [12,35].

In biotechnology, developing soft robotics components out of synthetic intrinsically disordered polymers (sIDPs) bio-inspired utilizing IDPs with a self-assembly/healing ability would permit the healing of both microscopic and/or macroscopic damage. In fact, a wide range of healing polymer mechanisms have been developed based on chemical principles [36]. While healing mechanisms have been incorporated in ceramics and metals, progress has primarily been made in self-healing polymers because of their high molecular mobility, which enhances healing and may yield the flexible disordered characteristics required for soft robotics [37,38].

The necessary requirements for an external trigger to start the healing action can be classified as autonomous or non-autonomous [37]. In autonomous systems, the healing mechanism is provoked by the damaging process itself [39]. These resemble biological processes that deliver healing agents. On the other hand, non-autonomous systems require an external stimulus for activating the healing process [40]. The stimuli can be light, heat, a chemical change, or mechanical force [41].

Moreover, self-healing mechanisms can be categorized as intrinsic or extrinsic [41]. Intrinsic self-healing materials rely on chemical groups and features inherent to the material, such as reversible chemical bonds, for their healing capacity. Extrinsic self-healing materials have a healing ability incorporated into the material that is not original to the material itself, such as healing agents encapsulated or in vascular systems or in microcapsules. Intrinsic healing mechanisms can be further subdivided depending on the interaction used to achieve healing, which can be physical or covalent [41].

The healing ability of our intrinsically disordered synthetic self-healing polymers relies on dynamic covalent interactions [39]. These dynamic covalent bonds can be reversible, and can break and reform, even though an excessive amount of energy is necessary for breaking them (150–550 kJ/mol) in comparison to physicochemical bonds such as hydrogen bonds (which have bond strengths of only a few kJ/mol) [42]. In fact, a usual approach in self-healing polymers is to use the dynamic covalent bonds as crosslinks to construct polymer networks [43]. Including a self-healing capacity through self-organization of synthetic intrinsically disordered polymers based on intrinsically disordered aggregating proteins will enable development of soft robots that are reliable and sustainable. Autonomous healing pushes us to obtain self-healing polymers with improved mechanical behavior.

The process of generation of the self-healing synthetic intrinsically disordered polymers, being interdisciplinary and intertwined, includes computational chemistry, computational chemical engineering, biotechnology, polymer chemistry and engineering, and material sciences and engineering, as well as soft robotics engineering. New synthesis mechanisms are provided herein for synthetic intrinsically disordered smart polymers with self-healing capabilities bio-inspired from intrinsically disordered proteins which are at the center of neurodegeneration. Furthermore, strategies for characterization of the new class of disordered polymers, and machining the bulk polymer structures into filaments for 3D printing, are discussed herein. The intrinsically disordered smart and self-healing synthetic intrinsically disordered polymers bio-inspired from IDPs which embrace structure-breaking amino acid residues’ functional groups do not exist in current literature. These presented mechanisms and strategies will provide the lacking intrinsically disordered polymers for soft robotics applications.

## 2. Synthetic Intrinsically Disordered Polymers 

### 2.1. Soft Robots

Much of the efforts in soft robotics were motivated by the demand for human-friendly co-robots that could be used for physical human and machine interactions [44]. Applications embraced robotic arms for industrial automation and pneumatically powered orthoses for human grasp assistance. The majority of robots are motorized or hydraulically regulated and rely on rigid materials for load bearing and actuation [44]. Such robots cause safety hazards in workplaces, and human and robot interactions have to be controlled and monitored. While soft robotic developments have been making great strides for more than ten years, an area of increasing attention is in soft bio-inspired robotics that provide new opportunities for producing engineered components, machines, and devices that can bridge the gap between natural organisms and robots [44]. However, many challenges exist in developing soft bio-inspired robots, including new material design and synthesis and new material-dependent system integration [44]. Some of the engineering principles for the development of soft bio-inspired robots are known, but new issues emerge, such as higher flexibility demands along with self-healing properties.

Soft robots, unlike hard-bodied robots, are currently composed of easily deformable materials such as gels, elastomers, and biological materials that usually have similar elastic and rheological properties of soft matter existing in nature [44]. Soft materials used in soft robots include urethanes, silicon elastomers, braided fabrics, hydrogels, hydraulic fluids, and gases. Among these, elastomers are very popular since they enable the usage of a broad range of desired viscoelastic and elastic properties within the materials [44]. These also have the advantage of safe interaction with biological organisms and the overall actuation of robots. We expect our intrinsically disordered polymers to be viscoelastic materials such as muscle, fat, and hydrogels. These may exhibit both viscous and elastic properties. Such characteristics allow for the potential of robot components to dissipate energy and maintain stable motion during loading. Common viscoelastic materials which exhibit elastic hysteresis are polyacrylates and polyurethanes. In addition, elastic strain limit is sensitive to characteristics that cause deformation in inhomogeneous modes, such as cracks, notches, and other stress concentrators. The intrinsically disordered synthetic polymers are expected to exhibit high fracture toughness due to their self-organization nature.

Robots hold great promise for amplifying human effectiveness in defense operations. Compared to human beings and animals, however, the mobility and manipulation capability of present-day robots is poor. In addition, design and manufacturing of current robotic systems are time-consuming and fabrication costs remain high. If these limitations were overcome, robots could assist in the execution of military operations far more effectively across a far greater range of missions. Synthetic intrinsically disordered polymers with self-healing and self-organization capabilities would minimize the financial burden and avoid injuries or even the more serious problems typically encountered in defense areas. Soft robots with greater flexibility could help in military operations, where level terrain and unobstructed areas are rare, whether as a fully intact robot or as smaller utility, such as a strap-on-arm with a soft arm gripper that could extend the reach, strength, and capability of what a person could do. The lightweight soft robotic materials are designed to overcome the challenges of traditional heavier exoskeleton systems, such as power-hungry battery packs and rigid components that can interface with natural joint movements. One of the advantages of these types of soft robotic materials is that one can design complexity into the highly flexible structure to simplify control requirements.

The synthetic intrinsically disordered polymers to be produced by our group will be used mostly in soft robotic applications. However, these smart and self-healing polymers can be modified to be used as building blocks in the construction industry and, due to their high flexibility, may have excellent applications in the face of natural disasters. An additional area where such highly flexible, smart, and self-healing intrinsically disordered polymers can be used is avoiding accidents that may occur in the aviation industry with as little damage as possible. These soft robotic application materials could also have medical uses, for example, as prosthetics, and could assist in the care of the elderly or disabled. Soft, inflatable robots could be stored in small spaces and easily transported. Self-healing, bio-inspired, smart intrinsically disordered polymers may be used in composite materials to afford them low weight, outstanding ease of application, and chemical stability in many environments.

### 2.2. Synthesis of Intrinsically Disordered Polymers

Synthesis of the new polymers is conducted using two different methodologies: 

#### 2.2.1. Addition of Structure-Breaking Amino Acid Functional Groups to the Chain Extender

**Scheme 1:**
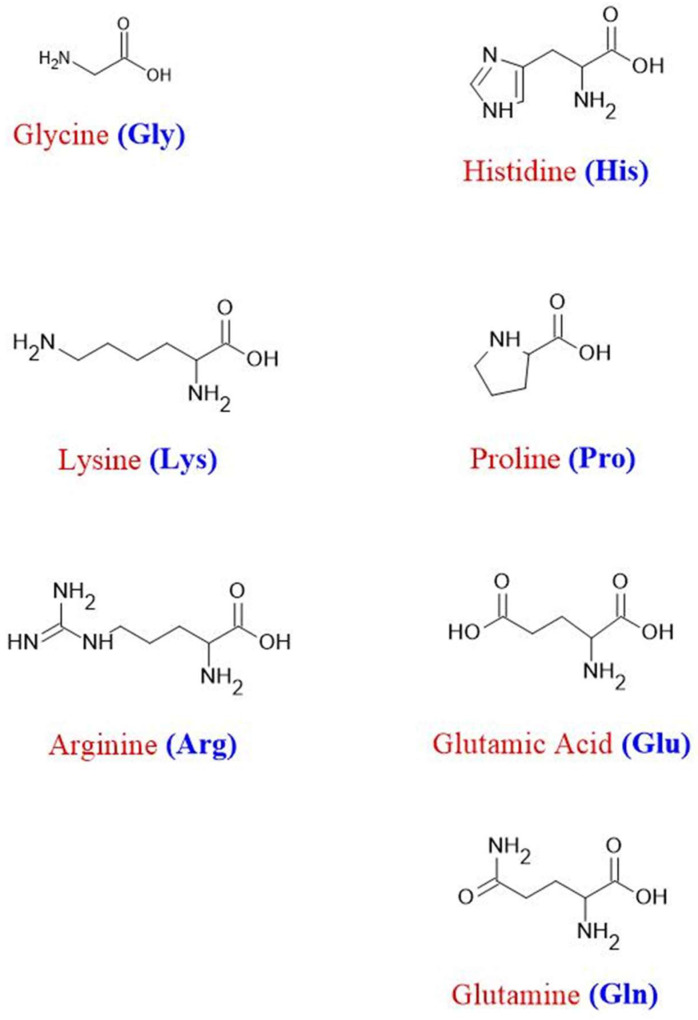
The chemical structures of seven structure-breaking amino acid residues.

The structure-breaking amino acid residues are shown in Scheme 1.

**Scheme 2:**
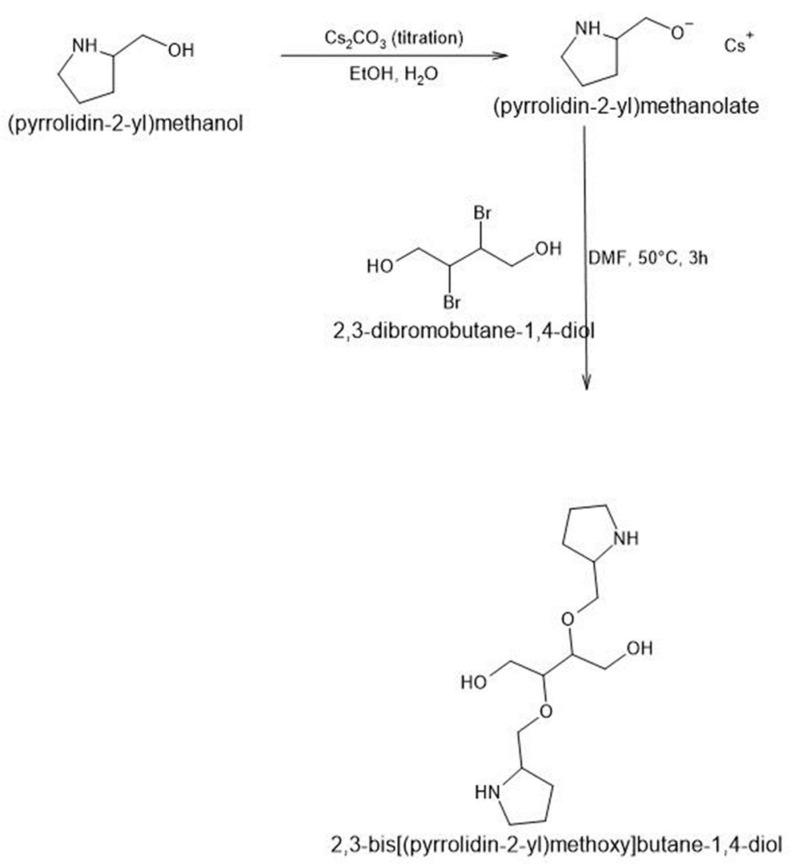
An example reaction mechanism using the functional group of proline for creating a new chain extender.

As an example, the reaction mechanism using the functional groups of proline for creating a new chain extender is provided in Scheme 2.

**Scheme 3:**
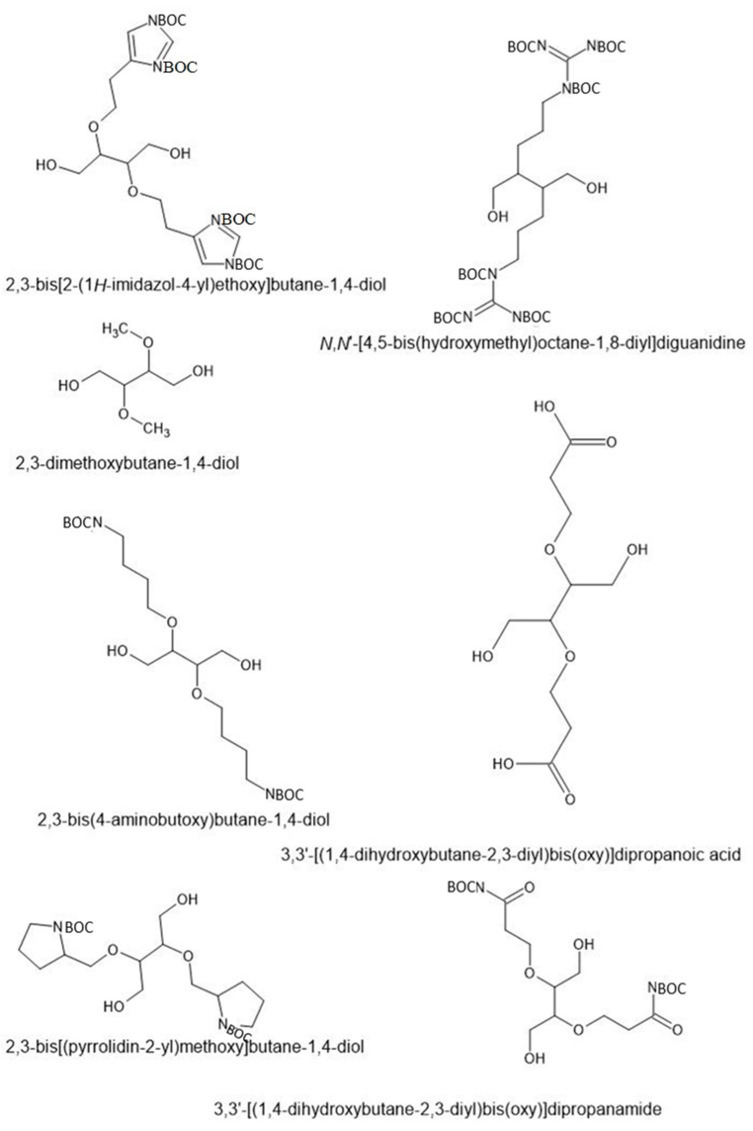
The new chain extenders using the functional groups of seven structure-breaking amino acid residues.

The new chain extenders will be synthesized using all seven structural-breaking amino acid residues’ functional groups. Specifically, the functional groups of amino acids will be modified with 2,3-dibromobutane-1,4-diol for obtaining the bio-inspired intrinsically disordered polymers. For this purpose (as shown in Scheme 2), the functionalization reaction will be carried out in two steps. In the first step, the acidic solution of the functional group salt will be titrated with an aqueous solution of cesium carbonate for activating the hydroxyl group. Next, the solvent will be evaporated and the obtained product will be stored in a desiccator for usage in the second step of the functionalization. In the final step, the obtained product will react with 2,3-dibromobutane-1,4-diol (1:1 molar ratio) under an inert atmosphere at 50 °C in an oil bath for 3 h. The resulting product is then attained by the extraction in the presence of dichloromethane (DCM). Tert-butyl hydrogen carbonate (BOC) will be utilized for protecting the amine groups. The synthesized new seven chain extenders are shown in Scheme 3. These new chain extenders will be used in the syntheses of a polyurethane-based new class of intrinsically disordered polymers.

#### 2.2.2. Synthesis of New Bio-Inspired Intrinsically Disordered Polyurethanes

**Scheme 4:**
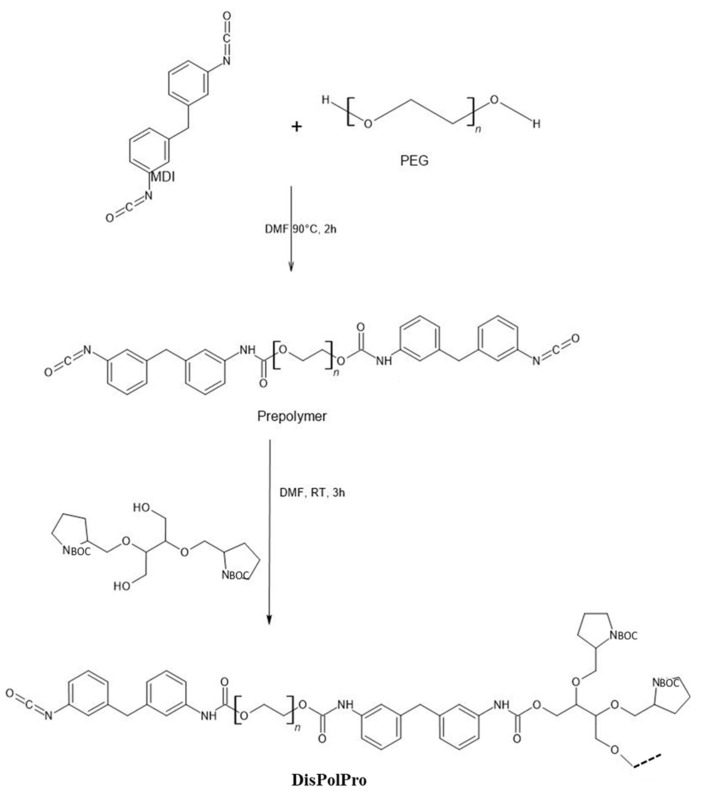
Polymerization reaction mechanism of the new polymers using the new chain extenders.

The polymerization reaction using the new chain extenders is demonstrated in Scheme 4. Here, 2,3-bis[(pyrrolidine-2-yl)methoxy]butane-1,4-diol is shown as an example. Note that similar reactions can be conducted for all chain extenders listed in Scheme 3.

**Scheme 5:**
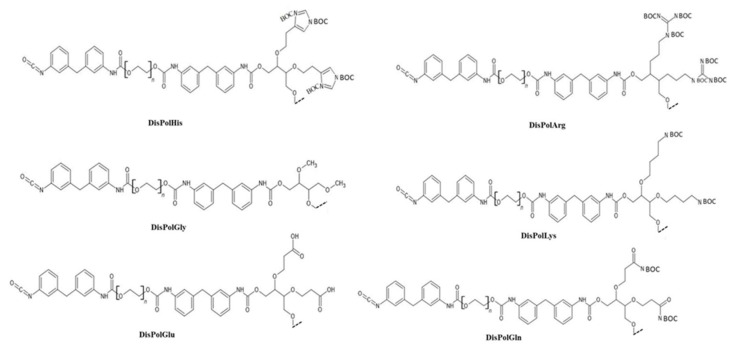
The new bio-inspired polymers and their chemical structures.

For obtaining bio-inspired intrinsically disordered polyurethanes, it is essential that the reaction is controllable. For providing more control, one can use a two-step polyaddition reaction so that one can obtain the desired polymer yield. In the first step of the reaction, PEG is used as a polyol and 4,4-methylenediphenyl diisocyanate (MDI) is utilized as diisocyanate. The polymerization reaction is carried out under inert atmosphere. Isocyanate (MDI) and PEG are dissolved in DMF at a molar ratio of 2:1 and mixed in an oil bath at 90 °C for 2 h at 400 rpm in the presence of a mechanical stirrer. In the second step of the reaction, the new chain extender is added to the prepolymer in the medium at a ratio of 1 molar, under constant stirring. This reaction takes 3 h at RT under inert atmosphere and constant stirring. The viscous solution obtained at the end of the reaction is dried in a vacuum oven at 60 °C for 36 h. The purification step is conducted by extracting the obtained polymer in the presence of chloroform solution. Scheme 5 presents the chemical structures of our bio-inspired new polymers. These new polymers may find useful applications in soft robotics arm gripper design and/or in bionic arm design.

#### 2.2.3. Preparation of Bio-Inspired Intrinsically Disordered Polyurethane Composites

One of the methods used to improve the mechanical properties of materials is to add particles to the matrix using appropriate proportions. In order to improve the mechanical properties of bio-inspired intrinsically disordered polyurethanes, carbon nanotubes (CNTs) particles are added to the polymer matrix at 1, 3, 5, and 7 weight ratios under constant mixing [45]. Vigorous mixing is performed to ensure that the particles are dispersed homogeneously in the matrix. Next, the morphological and mechanical properties of the obtained composites are examined. Accordingly, the polymer composite with the optimum properties is determined. These studies may be useful for obtaining flexible actuators and/or sensors.

#### 2.2.4. Functionalization of Polyols with Functional Groups of Structure-Breaking Amino Acids

**Scheme 6:**
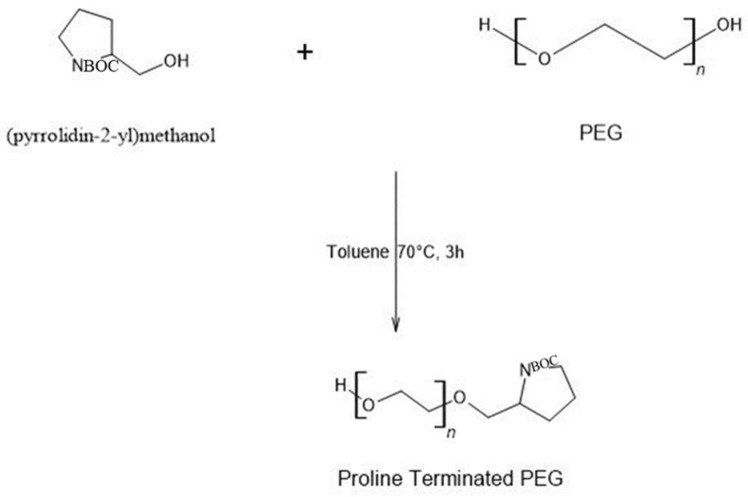
An example reaction mechanism for obtaining proline terminated PEG.

In addition, one can add the functional groups of seven structure-breaking amino acid residues to PEG for creating a second set of new polymers. Scheme 6 represents the reaction mechanism between proline’s functional group and PEG as an illustrative example.

**Scheme 7:**
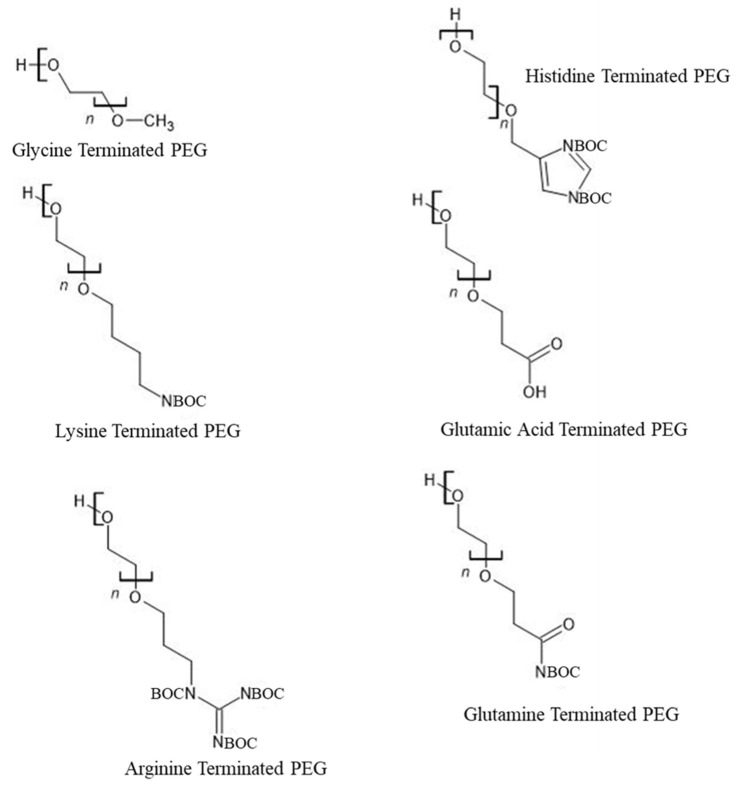
Obtained amino acid functional groups terminated PEGs. The new functional group terminated PEGs will be used in the polymerization reaction with isocyanate.

Using the reaction mechanism illustrated in Scheme 6, one can obtain glycine, lysine, histidine, glutamine, glutamic acid, proline, and arginine functional groups terminating PEG units. The chemical structures of the obtained products are depicted in Scheme 7.

#### 2.2.5. Polymerization Reaction of Functional Group Terminated PEGs with Isocyanate

**Scheme 8:**
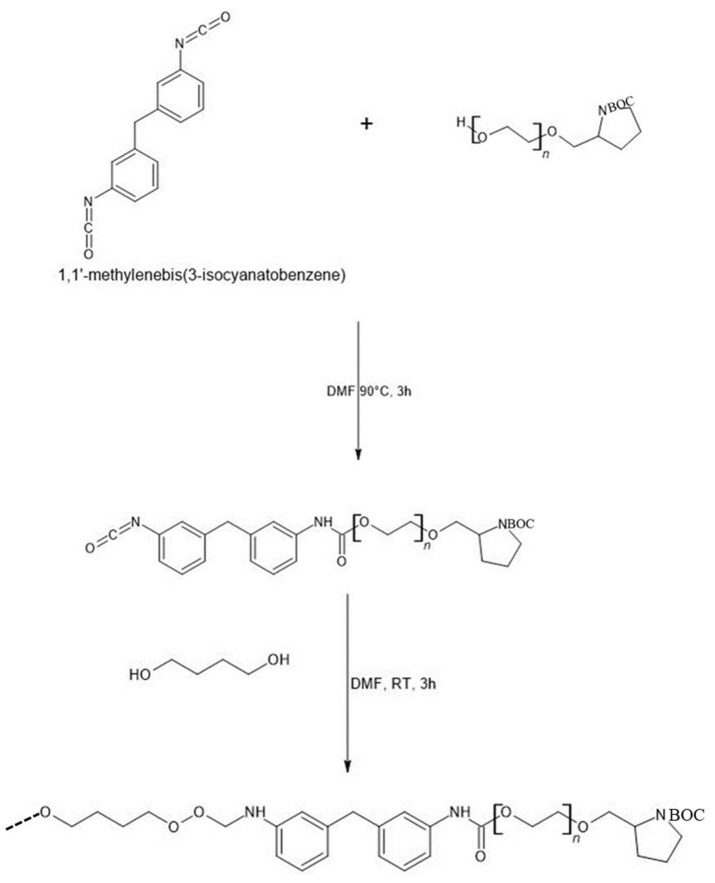
Polymerization reaction mechanism of the new proline functional group terminated PEG and diisocyanate.

Scheme 8 demonstrates an example of the obtained second class of polymers. This is only one example, and the reaction is conducted for all seven structure-breaking amino acid functional groups to yield a new class of polymers.

**Scheme 9:**
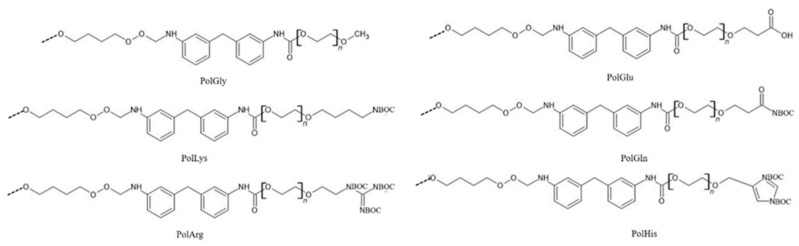
The new class of polymers using the seven structure-breaking amino acid residues at the end of the polymers.

Scheme 9 lists the chemical structures of obtained new polymers.

These new polymers may find application in the areas of soft robotic arm gripper and/or bionic arm design.

### 2.3. Characterization of New Class of Bio-Inspired Intrinsically Disordered Polymers

NMR spectroscopy can be used for the chemical characterization of resulting structures and polymers. ^1^H-NMR spectra will be recorded for all samples at room temperature on a 400 MHz Bruker AV400 spectrometer. All spectra will be corrected with phase and base line, and the solvent peak will be fixed before quantifying the degree of yield. In order to prove the chemical structure of products, Fourier transform infrared (FTIR) analyses in the wavelength range of 4000–400 cm^−1^ will be applied. Morphological characterization of bio-inspired intrinsically disordered polyurethanes and their composites are recorded by using scanning electron microscopy with an EDS-analyzer (SEM-EDS). In order to prepare the polymers before the analyses, specimens are dispersed in ethanol, fixed on adhesive carbon tapes, and sputter-coated with gold. The crystallinity of the polymers will be characterized by X-ray diffraction (XRD) measurements conducted on CuKα (λ = 1.5418 Å) radiation at 45 mA, 40 kV and Ni-filter.

Determining the glass transition temperature (T_g_) of polymers is important to better understand its usage conditions, while determining the melting temperature (T_m_) is crucial for improving process conditions. For this purpose, differential scanning calorimetry (DSC) analyses will be conducted under heating–cooling–heating conditions, such as heating the samples from −50 °C to 250 °C with a heating rate of 10 °C/min, followed by cooling them with a rate of 5 °C/min and heating again with a rate of 10 °C/min. Thermogravimetric analyses (TGA) of obtained polymers are performed using a thermal analysis system instrument under nitrogen flow with a heating rate of 10 °C/min. For better understanding the flow properties of bio-inspired intrinsically disordered polyurethanes, rheological analysis is performed using a rotational rheometer equipped with a 25 mm diameter parallel plate geometry. In order to characterize the samples, a stress-strain sweep test is recorded at a constant strain of 1% in a frequency range of 0.1–100 rad s^−1^ at room temperature and at significant temperatures. The recovery property determined using the rheology analysis is one of the most important parameters required to examine self-healing properties. To investigate the recovery time of bio-inspired intrinsically disordered polyurethanes, oscillation time sweep test will be carried out with strains varying between 250% and 0.5%, with the measurement conditions being every 2 min.

### 2.4. Machining of Bulk Polymer Structures into Filaments for 3D Printing Purposes

The obtained polymers will be granulated to the size of 4 mm ± 1 mm (diameter) at room temperature in order to have the material in the proper state to be extruded. For better processing of thermoplastic polyurethanes, pre-drying is required before loading into the extruder. The drying process is carried out between 2–4 h in air circulation ovens operating at 90–100 °C. Next, a double-screw extruder with different heating zones and the desired L/D screw ratio is used to obtain the filaments. The polymer granulate is extruded through a cylindrical nozzle (Ø2.9 mm) using certain pressure at determined T_m_ values of bio-inspired intrinsically disordered polyurethanes. At the exit end of the extruder, there is a cooling unit followed by a filament winding step. The filament diameter is controlled by an electronic caliper in order to achieve the desired filament size.

### 2.5. Mechanical Characterization of 3D-Printed Polymers

The advanced mechanical properties of 3D structures are some of the crucial parameters. In this context, tensile tests, compression tests, and dynamic mechanical analysis will be conducted respectively in parallel to the usage conditions of the products. Therefore, dog bone specimens are prepared by thinning to a total width of 14 mm over a 9 mm radius with a length of 45.5 mm and a thickness of 2 mm, with a gauge length of 17 mm and a gauge width of 7 mm. Testing is performed on a universal testing system with a 100 N load cell and custom self-tightening grips. Initial sample lengths will be measured as the distance between grips when testing. Stress is calculated as force divided by cross-sectional area. Samples are strained at a rate of 50 mm/min until torn. All samples will be measured five times.

It is of particular interest to examine the mechanical strength of materials with self-healing properties after healing. For this purpose, the self-healing samples are cut with a razor and then rejoined and held under dark conditions for 24 h. After the healing process, the same mechanical testing conditions are applied to the specimens. To investigate the thermomechanical behavior of 3D-printed polymers, a dynamic thermomechanical analysis (DMA) test will be performed on a DMA analyzer by decreasing the temperature. The glass transition temperature, T_g_, of the specimens is identified according to the peak of tanδ. For the compression test, cylindrical samples with a diameter of 13 mm and a height of 6 mm are printed. Experiments are carried out with a compression test device at a speed of 180 mm/min. Compression tests will be applied to five samples each. The average of the data obtained as a result will be taken and evaluated with histograms.

## 3. Conclusions

Here, we present new chemical reaction mechanisms and experimental designs for the synthesis of bio-inspired intrinsically disordered polyurethane polymers. These are a new class of polymers that are smart, flexible, and possess self-healing capacities. These new polymers can be manufactured by polymer and material industries for various purposes, including soft robotics applications, health industries, and defense industries. Moreover, we should mention here that these polymers are not biodegradable and therefore their application in medicine is limited. However, one could design a biodegradable new class of synthetic intrinsically disordered polymers using the full-length amino acid residues rather than only their functional groups for applications in drug delivery, organ transplantation, artificial organ design, and immune compatibility.

## Data Availability

Data presented in this study are available on request from the corresponding author.
